# Detecting Emotions through Electrodermal Activity in Learning Contexts: A Systematic Review

**DOI:** 10.3390/s21237869

**Published:** 2021-11-26

**Authors:** Anne Horvers, Natasha Tombeng, Tibor Bosse, Ard W. Lazonder, Inge Molenaar

**Affiliations:** Behavioural Science Institute, Radboud University, 6525 XZ Nijmegen, The Netherlands; natasha.tombeng@ru.nl (N.T.); t.bosse@ru.nl (T.B.); ard.lazonder@ru.nl (A.W.L.); inge.molenaar@ru.nl (I.M.)

**Keywords:** measurement of physiological arousal, electrodermal activity, skin conductance, education, learning, training, emotion, affect, multimodal data streams

## Abstract

There is a strong increase in the use of devices that measure physiological arousal through electrodermal activity (EDA). Although there is a long tradition of studying emotions during learning, researchers have only recently started to use EDA to measure emotions in the context of education and learning. This systematic review aimed to provide insight into how EDA is currently used in these settings. The review aimed to investigate the methodological aspects of EDA measures in educational research and synthesize existing empirical evidence on the relation of physiological arousal, as measured by EDA, with learning outcomes and learning processes. The methodological results pointed to considerable variation in the usage of EDA in educational research and indicated that few implicit standards exist. Results regarding learning revealed inconsistent associations between physiological arousal and learning outcomes, which seem mainly due to underlying methodological differences. Furthermore, EDA frequently fluctuated during different stages of the learning process. Compared to this unimodal approach, multimodal designs provide the potential to better understand these fluctuations at critical moments. Overall, this review signals a clear need for explicit guidelines and standards for EDA processing in educational research in order to build a more profound understanding of the role of physiological arousal during learning.

## 1. Introduction

Techniques to measure physiological arousal are on the rise because they can provide meaningful insights into humans’ mental and physical states [1]. One of the most commonly used methods to measure physiological arousal is electrodermal activity (EDA) [2]. EDA refers to the changes in the electrical properties of the skin due to sweat gland activity [3]. Developments in the field of wearable technologies and signal processing increased the accessibility and usability of EDA and provided researchers with the opportunity to explore the affordances of EDA measurements in different contexts [4]. This trend is also visible in the field of learning and education, where EDA can be used to measure arousal during learning and relate different teaching methods to students’ emotional responses [5]. Although there already is a long tradition in studying emotion during learning [6,7,8], only recently researchers started to use EDA to measure emotion in this context [9,10,11]. With physiological arousal, there is an opportunity to gain insight into emotions in an objective way due to its subconscious nature [12].

This systematic review aimed to provide insight into how educational researchers currently use physiological arousal, as measured by EDA. The EDA signal is complex and needs multiple processing steps, but no guidelines or standards are currently available in this field. Therefore, the *first objective* was to create an overview of methodological aspects of EDA applied in learning contexts. We started by reviewing which devices are used, how the EDA signal is processed, which features are extracted, and how the EDA signal is analyzed. The state of the art can possibly reveal implicit standards for EDA processing, which can be translated into explicit guidelines for future research. There seem to be two research strands within the emerging group of educational researchers who use EDA, one focusing on learning outcomes and one on learning processes. The *second objective* of this review was to examine existing empirical evidence of the interaction between physiological arousal as measured by EDA and learning outcomes and learning processes separately. Learning processes can be investigated with unimodal and multimodal designs. The unimodal approach views EDA as a single data stream and examines variations in EDA during the learning process. The multimodal approach explores the relations between EDA and different data streams to understand the learning process further.

To provide a theoretical background for this review, we first elaborate on the theoretical relation between emotion and learning and present the rationale behind the two objectives.

### 1.1. Theoretical Background of Emotion and Learning

The link between emotion and learning is widely investigated as emotion is integral to the learning process and influences students’ learning outcomes [6,7,13,14]. Positive emotions, such as enjoyment or pride, are positively related to learning, whereas negative emotions, such as frustration, tend to have a negative impact on learning [15,16,17,18]. Emotions are generally defined according to a *categorical* or *dimensional* perspective. Categorical theories divide emotions into different types, such as the six basic emotions: fear, anger, happiness, surprise, disgust, and sadness (Ekman, [19]). Each emotion has a distinct facial expression and action tendencies [20]. However, it has been argued that these basic emotions have little connection with learning [21,22]. Dimensional theories of emotion do have this association and describe emotions by two or three continuous factors. Two-dimensional models, such as the circumplex model by Russell [23], characterize emotion in terms of valence and arousal [20]. Arousal refers to the amount of physiological activation that occurs when an emotion is triggered [24], while valence indicates the pleasantness of an emotion ranging from positive or pleasant to negative or unpleasant [25]. The circumplex model has been extended into several three-dimensional models of which the Control-Value Theory is widely used in the field of education [25,26]. This model adds object focus (i.e., where the learner’s attention is focused on during the occurrence of the emotion) to valence and arousal [6].

An emerging body of research in the field of learning connects the arousal component of the dimensional theories with physiological measures [27]. Physiological arousal reflects complex subconscious responses of the body [9,12]. Research has shown that physiological arousal varies during the learning process: it increases when students initially see a difficult problem and fluctuates when calculating an answer to a problem or when a student receives feedback on an answer [27,28,29]. Arousal also has a direct influence on learning itself. A sufficient level of arousal is needed for students to pay attention to instruction or the exercises. The level of physiological arousal can be inferred from different modalities, such as heart rate variability (HRV), blood volume pulse (BVP), skin temperature, and EDA [2,26]. Previous research has recognized EDA as a reliable identifier of physiological arousal [12]. There is a substantial increase in the usability and accessibility of devices that measure EDA [3,30], and wearable technologies provide more opportunities to measure EDA in real-world settings [4,31]. EDA holds promise for measuring arousal during learning, as it represents a close to real-time activation that can possibly be linked to cognitive and emotional responses on a detailed basis [3,27,32]. Therefore, this review will look at the use of physiological arousal as measured by EDA in the context of education and learning.

### 1.2. Electrodermal Activity

EDA refers to the variation of electrical characteristics of the skin due to perspiration or sweat gland activity [3]. Eccrine sweat glands have thermoregulation as their primary function, but the glands located in the palmar (hands) and plantar (soles) regions are suggested to have a relation with behavior. EDA can be measured via endosomatic and exosomatic recordings [12]. When EDA signals are recorded without an external source of electricity, it is called the endosomatic method, which measures skin potential (SP) [12]. Measures of skin conductance are expressed in units of micro Siemens (µS) [12,33]. The exosomatic method applies a small external electrical current through electrodes placed on the surface of the skin to measure Skin Conductance (SC) [12,34]. This exosomatic method is used by researchers in various fields, and most studies in the learning and education field focus on skin conductance instead of skin potential [12]. Thus, we will focus on exploring skin conductance in this systematic review.

**Figure 1:**
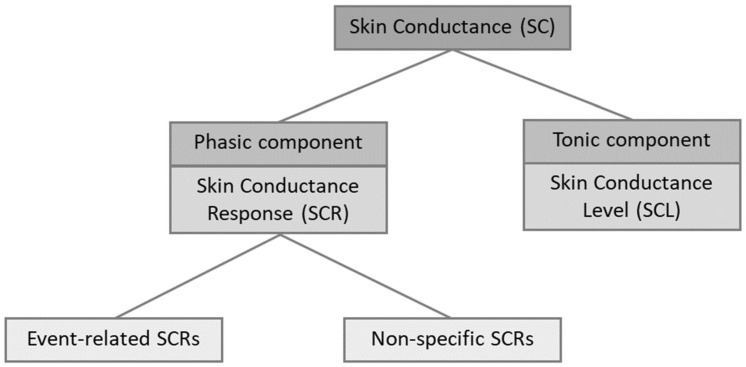
Components of skin conductance (adapted from [36]).

Skin conductance consists of a tonic and a phasic component (see Figure 1). The tonic component consists of slowly varying activity and is also called skin conductance level (SCL) [12]. Tonic activity generates a moving baseline per individual [35]. This signal is relatively stable within some seconds [3]. Phasic activity or skin conductance response (SCR) refers to the faster changing elements of the EDA signal [12]. Phasic activity represents a reactive response compared to the tonic components [36]. If SCRs are above a specific threshold, they can be referred to as peaks or spikes as well [35]. These peaks can be event-related or non-specific. Event-related SCRs can be attributed to a specific eliciting stimulus or task. Non-specific SCRs occur with no identifiable stimulus that elicits the response [35].

### 1.3. EDA: The Methodological Objective

The complexity of the EDA signal creates a need for multiple processing steps, such as removing noise and movement artifacts from the signal, extracting meaningful features, and applying appropriate analyses [3,4]. In other scholarly disciplines, EDA guidelines are available for processing, for example, fear, stress, or emotion-evoking stimuli [35,37,38]. However, such guidelines are not explicitly available for research on learning and education as approaches for processing EDA differ in this field. This unique context requires thoughtful consideration of how EDA can contribute to measuring constructs that are important for learning, such as emotion. Therefore, *the first objective* of this systematic review is to provide an overview of methodological aspects of EDA currently applied in the learning and education field. This overview can explicate possibly emerging standards for EDA processing which can be translated into future guidelines.

### 1.4. Physiological Arousal and Learning: The Empirical Objectives

As mentioned above, the emerging group of educational researchers who use EDA focus on two different research strands. The first one investigates the relationship between physiological arousal as measured by EDA and learning outcomes [39,40]. These learning outcomes generally refer to students’ performance on a learning task or a test. Previous studies found both negative and positive relations between physiological arousal and learning outcomes [36,41]. Moreover, studies in the field of collaborative learning found a relation between physiological synchrony and task performance [42,43]. Due to the differences in results, the *second objective* is to increase our understanding of the interaction between physiological arousal as measured by EDA and learning outcomes by looking into the empirical results of recent studies.

The second strand examines the interaction between physiological arousal as measured by EDA and learning processes. There is a clear trend of studies that specifically focus on what happens during the learning process visible in educational research [44,45]. EDA is viewed as one of multiple data streams, which results in both unimodal and multimodal approaches. The unimodal approach examines EDA as a single data stream during the learning process. This approach focuses on changes in physiological arousal during learning by investigating fluctuations and variations in EDA signals during the learning process. Previous research indicates that EDA varies during the learning process; for instance, EDA fluctuates when students work on a task, see a difficult problem, or get feedback [27,28,29].

The multimodal approach examines the relations between physiological arousal as measured by EDA and multimodal data streams to provide insights into the learning process. This approach is in line with the increased focus on the value of data to further understand learning in the field of multimodal learning analytics with specific attention to processes during learning [44,45]. The multi-componential nature of emotion points to individual differences in expression and experience of these emotions [6,46]. Therefore, when emotion is investigated with physiological arousal, this multi-componential nature needs to be addressed. Next to physiological responses such as physiological arousal as measured by EDA, two other types of emotional responses to personally meaningful stimuli are often distinguished: experiential and behavioral responses [47]. Experiential responses are the subjective personal experiences of emotion, which can be measured through self-reports. Behavioral responses refer to the visible behavioral reactions to an emotion, which can be observed from a person’s posture and facial expression [47]. Other measures of physiological responses besides EDA can include electroencephalography (EEG), electromyography (EMG), electrocardiography (ECG), heart rate, and skin temperature. There is an opportunity to use a multimodal approach by combining physiological, behavioral, and experiential measurements [2,48,49]. Combining multiple data streams of different modalities has the potential to gain a deeper understanding of students’ learning processes [31]. For example, EDA data, facial expression detection, and self-reports can be combined to identify episodes of high arousal during learning and give meaning to these episodes [10,46]. The use of multimodal data can help overcome constraints related to the use of a single measurement. For example, self-report data can be modified by participants due to the awareness of their environment [46]. Measuring emotion from a single modality can result in partial inferences by overlooking other, more subconscious aspects of emotion [50]. Therefore, the *second objective* was extended to provide insights into how physiological arousal as measured by EDA varies during the learning process (unimodal) and to identify combinations of physiological arousal as measured by EDA with multimodal data streams to understand learning processes (multimodal).

### 1.5. This Study

A recent review by Posada-Quintero and Chon [4] described innovations in EDA data collection and signal processing by synthesizing the results of studies across a wide range of contexts. The present literature review went beyond the data collection and signal processing phases and additionally focused on the features that can be extracted from the EDA signal and empirical results of studies. Moreover, this review focused specifically on the complex field of learning and education. Every type of education was considered in this systematic review: primary education, high school, university, and adult education. We focused on learning activities in every subject, from foundational knowledge and skills (i.e., language and mathematics) to more advanced learning content. Moreover, both direct instructional methods and more student-centered and self-directed ways of learning, such as critical thinking and inquiry-based learning, were considered. This systematic review aimed to provide an overview of research regarding the measurement of physiological arousal through EDA in educational settings. Towards this end, two objectives were addressed:

(1) Methodological objective: Provide an overview of methodological aspects of EDA and investigate implicit guidelines and standards for EDA processing in educational research.
(2) Empirical objectives:
  - Examine existing empirical evidence of the interaction between physiological arousal as measured by EDA and learning outcomes
  - Examine existing empirical evidence of physiological arousal as measured by EDA during the learning process
    (a) Examine how physiological arousal as measured by EDA varies during the learning process (unimodal)
    (b) Examine combinations of EDA with multimodal data streams to understand learning processes (multimodal)

## 2. Research Method

### 2.1. Search and Inclusion of Studies

Literature searches were performed in July and August 2021 in Web of Science (WoS), Education Resources Information Center (ERIC) Digital Library, and Google Scholar to identify relevant research articles and conference papers. These searches were extended by perusing the online proceedings of the following conferences: Artificial Intelligence in Education (2011–2021), Learning Analytics and Knowledge Conference (2011–2021), Educational Data Mining (2009–2021), Conference on User Modeling, Adaptation and Personalization (2009–2021), and the International Conference on Multimodal Interaction (2009–2020). Snowballing of key publications was applied to obtain the completest initial set of studies as possible. Only studies written in English were considered.

To find potentially relevant studies, three main areas of interest were identified: physiological arousal, learning, and emotion. Strings of multiple keywords were created for each area. Physiological arousal was the overarching term used in this review, which can be measured in different ways. As this review aimed to map the use of EDA in education, and since EDA is also referred to in the literature as skin conductance or galvanic skin response, we combined these four keywords in the query “physiological arousal OR electrodermal activity OR skin conductance OR galvanic skin response”. The keywords education and training were added to form the search string “learning OR education OR training” within the concept area of learning. Likewise, searches for studies on emotions were performed with the query “emotion OR affect”. These three queries were combined with the Boolean operator AND.

**Figure 2:**
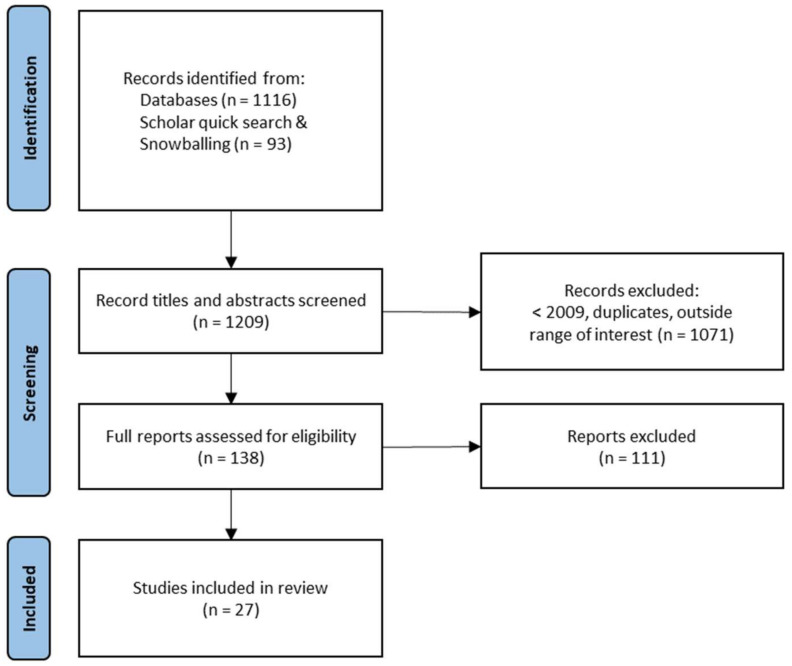
Study Selection and Inclusion Process (adapted from [51]).

The database searches resulted in 1116 studies (WoS = 856 and ERIC = 260). The conference proceedings, a quick search of the first 15 pages of results in Google Scholar, and snowballing yielded an additional 93 studies, bringing the total to 1209 studies. As shown in Figure 2, screening the studies’ titles and abstracts reduced the set of potentially relevant studies to 138. Excluded studies were outside our range of interest (e.g., fear learning and machine learning), duplicates, or published before 2009. The starting year 2009 was chosen because of technological advancements in wearable physiological arousal sensors and to extend the recent review by Posada-Quintero and Chon [4], who also used this starting point.

Next, the 138 potentially relevant studies were assessed against the inclusion criteria. To be eligible for inclusion in this review, a study had to meet the following inclusion criteria: (a) report empirical results, (b) measure EDA or (galvanic) skin conductance, (c) in the context of education, learning, or training, (d) investigate the relationship between physiological arousal and emotion, and (e) investigate the interaction between physiological arousal and learning. To assess if studies were eligible for inclusion, the full text was read by two independent raters. Disagreements regarding inclusion versus exclusion of a study were resolved through discussion. After applying the inclusion criteria, 27 studies were included in this review (see Table 1 for study characteristics).

### 2.2. Study Feature Coding

The included studies were analyzed according to the main objectives of this review. For the first objective (methodological aspects), the *devices* used to measure physiological arousal and their corresponding sampling rates were extracted from the primary studies. Information about the EDA *signal processing*, including filtering (noise removal), cleaning (exclusion of movement artifacts), and normalization methods (as a way of accounting for individual differences), was extracted as well. The authors also coded whether the primary studies used *baseline measurements*. If so, information about the activity during the measurement, length of the measurement, and further utilization of the baseline were extracted. Finally, the *features* that can be extracted from the EDA signal and the *extraction methods* used were derived from the studies.

For part I of the second objective, empirical results of the interaction between physiological arousal as measured by EDA, and *learning outcomes*, such as students’ performance on a task or test, were obtained. Part II of the second objective focused on the *learning process*, so empirical results of how EDA *varies* and *fluctuates* during the learning process were extracted. Moreover, empirical results of combinations of EDA with experiential, behavioral, and other physiological responses measured by *multimodal data streams* were extracted. Experiential responses included subjective experiences of emotions which can be measured through self-reports. Behavioral measures included eye-tracking and facial expression detection. Other physiological measures besides EDA included EEG, EMG, ECG, heart rate, and skin temperature.

## 3. Results

The results are divided into two main sections. The first section addresses the first objective of identifying methodological aspects used in recent studies on physiological arousal as measured by EDA and learning. The second section focuses on the second objective, which includes the empirical findings of this review, namely the interaction between physiological arousal as measured by EDA and learning outcomes, variations in physiological arousal as measured by EDA during the learning process, and combinations of EDA with multimodal data streams. Table 1 shows the study characteristics of the included studies.

### 3.1. Methodological Aspects of EDA

This section provides an overview of methodological aspects of EDA in educational research. We discuss measurement devices, the processing of the EDA signal describing filtering, cleaning, and normalizing of the signal, baseline measurements, different features that studies extracted from the EDA signal, and finally, the methods used to extract these features. Table 2 shows an overview of these aspects.

#### 3.1.1. Devices to Measure EDA

Currently, many different devices are available to measure EDA with different sampling rates. The sampling rate is the number of samples obtained in one second and is indicated in hertz. Generally, a sampling rate above 10 hertz is considered sufficient to measure EDA [72]. Braithwaite et al. [35] recommend a sampling rate of 1 to 5 samples per second for longer-term measurements (1 to 5 hertz). However, when the signal is divided into tonic and phasic components, a sampling rate of at least 4 to 8 hertz is needed [3]. An advantage of even higher sampling rates is that they ensure an event can be accurately represented in the measurements [35]. Devices used in laboratory settings mostly have a high sampling rate. Generally, wearable and wireless devices that can be used in authentic settings more easily have lower sampling rates [72].

As shown in Table 2, the Empatica (E4 and E3) was most frequently used in the included studies (*n* = 11) and has a sampling rate of 4 hertz. For longer-term measurements, this sampling rate is sufficient [3], but higher sampling rates are recommended in the literature [72]. Biopac (MP150) and Biosemi (Active 2) have a higher sampling rate of 1000 hertz and were both used in three studies. These devices are mostly used in laboratory settings and are harder to use in authentic settings due to their size. The Q-sensor (2.0; *n* = 2) resolves this issue as it is wearable, but it only has a sufficient sampling rate for longer-term measurements (8 hertz). The BodyMedia device (*n* = 1) and Shimmer3 GSR+ (*n* = 1) have a 32-hertz and 51.2-hertz sampling rate respectively. They both meet the sampling rate criteria and are wearable and thus easier to use in classroom studies.

The placement area of the electrodes in these devices differs as well. As eccrine sweat glands are most prominent on the palmar and plantar regions, EDA recordings are most promising when measured on the hands or soles. None of the included studies measured EDA from the soles [3,12]. Eight studies placed electrodes on the fingers by using Biopac, Shimmer, or Biosemi. Most often, these electrodes were placed on the middle phalanges of the index and middle finger. When electrode placement on the hands or soles interferes with the task, measurement through the wrist is a viable alternative, but the quality of the signal is lower due to fewer eccrine glands [12]. The studies that used Empatica and Q-sensor measured EDA from the wrists. The BodyMedia was used on the left upper arm, which is not the preferred choice to measure EDA because of the lack of eccrine sweat glands in that region. In general, the non-dominant hand was most used to measure EDA (*n* = 14), but not all studies reported which hand they used. Measurements on the non-dominant hand side are preferred because the chance of movement is lower, and the dominant hand can be used for the task [12].

#### 3.1.2. Processing EDA

The included studies differed on how the EDA signal was processed. Most studies (19 out of 27) first separated the signal into tonic and phasic components, but there was no consensus on which component to use. Six studies used both tonic and phasic components to further analyze the EDA signal [52,53,56,58,61,69]. Additionally, six studies used tonic components, i.e., skin conductance level (SCL) only [39,40,49,55,63,65], and seven studies used phasic components, i.e., skin conductance response (SCR) only [33,36,50,60,64,66,68]. Six studies used the raw EDA signal for feature extraction and analysis, and two studies provided no information on processing [54,62].

#### 3.1.3. Signal Processing: Filtering, Cleaning, and Normalization

Regarding the processing of the EDA signal, nine studies did not report information about cleaning or filtering [40,46,53,57,60,61,62,67,68].

*Filtering* was used to exclude noise from the EDA signal in ten studies. Multiple studies used a low-pass filter to eliminate high-frequency noise from the EDA signal [54,58,64,69]. These studies used different cut-off frequencies, ranging from 1 to 5 hertz [58,69]. Other studies combined a low-pass Butterworth filter with a high-pass filter (cut-off at 0.01 hertz) or only used a high-pass filter [39,63]. The actual filtering was done with different tools, such as the Matlab toolkit Ledalab [49,58], the EDA-Explorer tool [56], Brain Vision Analyser Software [66], or Acqknowledge [59]. Next to noise removal, studies used down-sampling to compress the EDA signal for easier processing. Cowley et al. [58] down-sampled from 32 to 16 hertz, and both Fox [49] and Meer et al. [66] down-sampled their 1000 hertz sampling rate to 40 hertz and 10 hertz, respectively. This down-sampling was done when the data set was too large to process [73].

Eight studies *cleaned* their signal, which implies the exclusion of movement from the physiological signal. Three of them used both filtering as well as cleaning [50,59,63]. A prevalent form of cleaning is manual and visual detection of movement artifacts [55,64]. Khan et al. [36] removed noise by calculating the total movement using L2-norm calculation (sqrt(x^2^ + y^2^ + z^2^)). They then removed the top and bottom 5% of the standard deviation of the total movement from the signal. The removed data were replaced with the mean EDA of the remaining data [36]. Another method to clean the signal is to use interpolation, where movement artifacts are replaced with new data based on the existing signal [52]. Machine learning can also be used to detect these artifacts. For example, Collins et al. [58] used machine learning (support vector machine) with a classification accuracy of 95.67% to detect movement artifacts. Finally, an extra signal measured by an accelerometer built-in in some EDA devices was also used to detect movement artifacts [36,59,71].

Six studies *normalized* their EDA signal to account for individual differences in the EDA signal. Two of these studies combined normalization with cleaning [52,71], and one study combined normalization, filtering, and cleaning [64]. Both studies of Villanueva et al. [70,71] used normalization through range correction. This technique takes into account the minimum and maximum amplitude levels. Another way to standardize the signal, as used by three studies, is using z-scores, which are calculated using the mean and standard deviation of the whole population [52,63,64]. Apostolidis et al. [54] also normalized their physiological signal but did not elaborate on how they have done that. Another way to account for individual differences in the EDA signal is by using a baseline measurement, used by 13 other studies, as we will describe below.

#### 3.1.4. Baseline Measurement

Normalization of the EDA signal can also be done by using a baseline to correct for variation between individuals (*n* = 13) [12,30]. In the literature, the tonic component of EDA is referred to as the baseline as well [3,35], but here we discuss the specific measurement of a baseline.

In five studies, the baseline was recorded while participants watched a video [49,52,55,65,69]. These videos ranged from relaxing nature videos to neutral videos (e.g., a fishbowl) [61,65,69]. Other ways were using breathing exercises with audio [68] or collecting a baseline during resting time [49]. A more advanced approach that included different tasks and recovery periods was used by Blikstein et al. [55]. Five other studies reported collecting the baseline while no specific activities were done [46,58,61,66,70]. Other studies used the learning sessions to calculate an a posteriori baseline [40,53,58,65]. Cowley et al. [58] collected the baseline repeatedly between learning tasks to account for possible changes in the baseline due to the task. Hoogerheide et al. [40] used the average of EDA during two learning tasks and divided it by two. Antoniou et al. [53] and Mason et al. [65] used the first learning session as the baseline recording.

The length of the baseline recording varied from a minimum of 30 s [69] to a maximum of 22 min [55]. Most studies used a 4 to 5-min baseline recording [49,58,61,65,68]. See Table 2 for an overview of the lengths of all baseline measurements.

How the baseline measurements are utilized differs: some researchers used the baseline in their analysis [40,58,66,68]. Four studies used baseline measures to calculate different features of EDA. Harley and colleagues [46] addressed arousal compared to individuals’ baseline and indicated whether it was higher or lower than the baseline. Two other studies computed the difference between arousal during baseline measurement and arousal during a learning task [49,65].

Baseline measurements can also be used to normalize or standardize data before further analysis as another way to account for individual differences in the EDA signal (see Section 3.1.3 for other ways). A group of researchers normalized their data with a user-dependent model that used individual participants’ baseline [46,61]. Others used a similar technique by computing the difference between skin conductance level (SCL) during a learning task and SCL during baseline collection [55,65].

#### 3.1.5. Features of EDA

There is a wide variety among the studies of which features are used. As shown in Table 3, descriptive features such as the mean, standard deviation, minimum, maximum, and range were extracted in 19 of the 27 studies. The most commonly used feature (*n* = 18) is the *mean EDA* of an individual participant [36,39,40,46,49,50,52,53,54,56,57,58,59,63,65,67,69,70,71]. The mean EDA is the average of EDA in a specific time period, but how this is calculated differs between studies. As mentioned above, the EDA signal consists of two components, which are phasic (SCR) and tonic (SCL). Several researchers focused specifically on the skin conductance responses (SCR) and calculated the average SCR during the entire learning session [36] or a specific phase in the learning process (such as a predefined task) [50,69]. Others focused on SCL and calculated the average of SCL of the whole learning session [56,65] or the average of SCL of a specific phase in the learning process (such as a predefined task) [40,49,65]. Segmenting the learning period is also a commonly used approach; the average of SCR and/or SCL is then calculated over a specific period of time, often one minute [58,60,61,64]. Three of these studies did not report why they chose the time window of one minute [58,60,61], but Malmberg et al. [64] explained they focus on event-based SCR, which occurs from 3 (low arousal) to 25 (high arousal) times a minute. Carroll et al. [39] calculated a weighted mean (root-mean-square) in their study. The root-mean-square embodies the mean SCL per participant weighted by the variability in the signal [39].

Seven out of 27 studies did not extract tonic (SCL) and phasic (SCR) components from the signal, but extracted features from the raw signal [46,53,57,59,67,70,71]. One study used the average of raw EDA data during a lecture [57], and two studies during specific tasks [57,67].

Other descriptive statistics were the *standard deviation, minimum, maximum, percentile features*, and *range* [46,56,57]. In a study on classifiers to detect emotional components during learning, the standard deviation of SCL is used as input for the classifier [56]. To train their classifier, they used minimum, maximum, and the 20th, 80th, and quartile deviation (25th and 75th percentile) of SCL. Harley et al. [46] used the range of the EDA signal per individual participant.

Another approach is SCR peak detection, where a threshold is used to define whether an increase in EDA is classified as a peak. This threshold varies between studies, but a threshold of 0.05 µS is most commonly used, which is also a standard in older EDA sensors [33,64,69]. With newer EDA sensors, a threshold of 0.01 µS is more common [68]. Many features can be extracted from SCR peaks. The *number of SCR peaks* is a commonly used feature, counting the number of peaks in the whole learning phase [69] or a segment of the learning phase (usually one minute) [61,64]. Another feature is the *frequency of SCR peaks*, calculated by dividing the sum of SCR peaks by the duration of a predefined phase, for instance, a learning task [64,68]. Additionally, the *sum of SCR amplitudes* is used, which is calculated by adding up the amplitudes of all significant (above threshold) SCRs [66]. Moreover, the *onset* of an SCR, i.e., the start of a peak, is used by Pijeira-Diaz and colleagues [33]. The *latency***,** the time from the onset of a stimulus to the onset of the response of skin conductance amplitudes, is used by Meer et al. [66].

Harley and colleagues [61] calculated a *standardized SCL score* between 0 and 1 for each participant. The minimum value of skin conductance extracted from the baseline measurement and the maximum value from the entire session was used based on the following formula: ((Standardized EDA Response = (EDA value − minimum value)/(maximum value − minimum value)) [12]. To calculate the mean SCL level, the average of the standardized scores was used. A similar approach was used by Blikstein et al. [55], but they used the difference between the skin conductance level and the baseline measurement. Hardy et al. [60] used a binary approach to indicate if a student showed a skin conductance response.

#### 3.1.6. Feature Extraction Methods

There are different approaches for analyzing EDA data and the extraction of features, as shown in Table 2. Initially, mostly *manual* hand-extracted trough-to-peak (TTP) methods were used for analysis [74]. This peak detection method indicates SCR amplitudes as the difference of the value at its peak and the preceding trough [73]. Some studies used a non-specified manual approach to extract features from the EDA signal [39,54,59,68].

A recent literature review described the shift from manual scoring of EDA data to automated EDA scoring, such as *the tonic—phasic decomposition of EDA*. [4]. Different toolboxes and algorithms can be used to decompose the EDA signal. Depending on which device was used to measure EDA, the accompanying software can extract features and sometimes decompose the signal as well. The most used toolboxes are Matlab-based. Nine studies used the Ledalab toolbox for their feature extraction [33,36,49,52,58,60,64,66,68]. Two analysis methods to extract tonic and phasic components can be used within the Ledalab software, Continuous Decomposition Analysis and Nonnegative De-convolution [73]. It is unclear in most of the included studies which method they used. Other tools are used as well, such as the Acqknowledge software [69], the Biograph Infiniti software [55], Augsburg Biosignal Toolbox (AubT) in Matlab [46,62], Neurokit with Makowski’s algorithm [50], and the cvxEDA-tool [56].

A challenge with multimodal approaches is how to *synchronize* data streams. To ensure the synchronization of multimodal data, tools can be used to capture different data streams simultaneously. Studies used different methods to ensure synchronization, like Observer [69]. Another way to ensure that the data is aligned is to ask participants to press a button each time they start and finish a task. This results in log files with timestamps and button-press stamps, which can be used for synchronization [50].

### 3.2. Empirical Results

This section focuses on the second objective of this review. First, we address the interaction between physiological arousal as measured by EDA and learning outcomes. Next, we focus on how physiological arousal as measured by EDA varies during the learning process (unimodal), and lastly, on combinations of EDA with multimodal data streams to understand learning processes (multimodal). See Table 4 for an overview of all empirical results.

#### 3.2.1. Learning Outcomes

Fourteen studies looked at the relationship between physiological arousal as measured by EDA and learning outcomes. In total, nine studies found a significant relationship between learning outcomes and physiological arousal as measured by EDA. However, three studies reported no significant relations between physiological arousal and participants’ performance on a mathematics test [68], engineering tasks [71], and a problem-solving task [40]. Nine studies used the *performance of participants on a task or test* to analyze this relationship. Some studies used exam scores or test performance as an outcome measure; for example, Pijeira-Díaz et al. [33] indicated arousal episodes (periods of a certain amount of arousal) by categorizing arousal into three levels (low, medium, high). The number of these arousal episodes (frequency) during an exam significantly correlated with learning measured by exam grade (*r* = 0.66, *p* = 0.02) (*r* stands for the correlation coefficient [75]; *p* is the probability value [76]) [33]. Khan et al. [36] also suggest a potential association between physiological arousal and exam performance. Their analysis established links between physiological arousal, skin temperature, and performance (*r* = 0.45, *p* < 0.05), and a weak positive relation between physiological arousal and performance (*r* = 0.16, *p* < 0.01). Mason and colleagues [65] investigated arousal during multiple-text comprehension tasks and found that the higher the increase in arousal, the lower students’ performance. At a micro-level, Ahonen et al. [52] examined whether arousal differed when students passed or failed on a collaborative programming task (correct or incorrect code). They found that incorrect code induces arousal around the moment of the event itself and that students showed decreased arousal before the correct code was submitted. *Learning gain* was also used as an outcome measure by two studies. Carroll et al. [39] found a significant correlation between science learning and changes in physiological arousal between two test times (*r* = 0.141, *p* < 0.05). Hardy et al. [60] showed that students had greater learning gains when they exhibited a skin conductance response after a specific behavioral event compared to students who did not (*t*(36) = 2.58, *p* = 0.014) (*t* or t-value is the size of difference relative to the variation in the data [77]).

Other studies also investigated whether physiological arousal can *predict* learning outcomes. Preliminary results of linear regressions by Harley et al. [46] showed that SCR is a significant predictor of the performance of medical students on a diagnostic task (*R*^2^ = 0.33, *p* < 0.05, *β* = 0.58, *p* < 0.05) (*R*^2^ or R squared is the coefficient of determination [78]; *β* or Beta stands for the probability of Type II error [79]). However, SCL was not related to learning in this study [46]. Li and Lajoie [50] also found that the phasic component of EDA was a significant predictor of performance, but then on low difficulty tasks in aviation training (*F*(1, 17) = 7.41, *p* < 0.05, *std β* = 0.55) (*F* or F-value is the ratio of two variances [80]; *std β* is the standardized beta coefficient [81]). Phasic EDA accounted for 30.4% of the variance in performance on tasks with an easy difficulty; for harder tasks, no significant predictive model was found. Cowley et al. [58] found that the tonic component of EDA predicted students’ learning gain (calculated by the difference between post-test and pre-test), the general level of tonic arousal was increased for participants with better learning. A similar result was found by Fox [49], the change in SCL over time predicted performance. Participants who had high arousal at first, which decreased over time, performed better than participants with little variation in their SCL.

Self-report measures are also used to address learning. Collins et al. [56] had participants indicate moments they experienced learning themselves and trained a classifier with EDA data to indicate these. They found that the average accuracy of this classifier was 83.66%. Cowley and colleagues [58] also used a self-report measure to address learning (additional to pretest-posttest measures). The phasic component of EDA was negatively associated with self-reported learning.

#### 3.2.2. Unimodal Approaches to Studying Learning Processes

Twelve studies investigated how physiological arousal as measured by EDA changes during the *learning process*. Seven of them indicated a change of EDA across different learning activities. For example, Antoniou et al. [53] compared EDA during a baseline and learning session in virtual reality and found a significantly higher EDA level during learning. An increase in arousal levels was also found by Meer et al. [66]; these levels were higher when active learning was introduced while oral reading and the latency of EDA was longer when students were oral reading in comparison to silent reading [66]. Geršak et al. [59] found that the mean EDA level of a group of children who engaged in a movement-based method to learn geometry was significantly higher than in the non-physically active group [59]. Other researchers found that SCL increased significantly during educational clips [69] and lessons [39]. A U-shaped EDA curve was found by Blikstein et al. [55] during physics tasks like building a bridge or tower. Participants’ physiological arousal increased when starting the task, dropped during the first part, and increased again at the end. Two studies found a significant decrease in EDA during learning. Irfan et al. [63] found a decrease in students’ SCL when they worked with virtual interactive materials on electrical circuits compared to a pre-recorded video. Likewise, Villanueva et al. [71] found a decrease in two of three engineering tasks, which may have occurred because of the difference in task type; a multiple-choice task showed a decrease, and hand-written tasks did not. Another study examined the occurrence of different arousal levels during learning by categorizing it into three categories: low, medium, and high arousal [33]. They found that low arousal was the most dominant state (on average 60%), medium arousal occurred in 24%, and high arousal in 17%. The low arousal state persisted the longest as well (on average 151 s).

However, significant differences in EDA levels during learning were not always found. Van Bruinessen and colleagues [69] did not find a significant change in SCR while comparing different educational episodes. The results of Hoogerheide et al. [40] also showed no difference between baseline (calculated by summing the average EDA during the first and second learning task and dividing this by 2) and EDA level during problem-solving in an electric circuit task.

#### 3.2.3. Multimodal Approaches to Studying Learning Processes

Physiological arousal can be combined with experiential measurements, behavioral measurements, and other physiological measurements, such as self-reports, facial expression detection, heart rate, and EEG. Almost all studies included in this review (*n* = 24) used multiple modalities and data streams in their studies (see Table 4), but seven studies did not use these to examine their connections with EDA and only looked at outcome measures. The results of studies that did examine connections with EDA are summarized below, organized by the type of complementary measurement.

**Experiential Responses.** Most multimodal studies used self-reports to gain additional insights into participants’ emotions (*n* = 14). These self-report measures can target *multiple emotions* or single emotions. Three studies used a self-report on multiple emotions, but their results were different. Harley and colleagues [46] concluded that there was no tightly coupled relation between physiological arousal and self-reported emotions. They used 5-point Likert scale questions about 19 separate emotions filled in five times during 90-min learning sessions to collect self-reported experiential data. They found an agreement of only 41.3% between physiological arousal and self-report data. The highest agreement was found between boredom and low arousal and neutral and low arousal. A possible explanation is that Harley et al. [46] used 10-s windows around the self-report event to extract features, which can lead to underestimation of meaningful EDA. On the contrary, correlations were found by the two studies by Villanueva and colleagues [70,71]. The first study used a questionnaire for discrete emotions and showed a positive correlation between the self-reported emotions and EDA (*r* = 0.44, *p* < 0.05) [71]. The second study used a different questionnaire for discrete emotions, using the dichotomous distinction of negative versus positive emotions [70]. Moderate correlations were found between EDA and negative emotions and EDA and positive emotions. Only in one learning session, they found a significant relation between EDA and negative emotions (*r* = −0.56, *p* < 0.05). These studies both used mean EDA over a whole learning session, which could have resulted in overestimation of the signal.

Some studies used a self-report measure focused on a *specific emotional state*, such as anxiety. Strohmaier et al. [68] asked participants twice to fill in a questionnaire on mathematics anxiety with a 4-point Likert scale. Their self-reported anxiety was not associated with physiological arousal (*r* = 0.06, *p* = 0.63). Meer and colleagues [66] also used a 4-point Likert scale questionnaire on state and trait anxiety. They found a significant association between the sum of SCR amplitudes and trait anxiety (*r* = 0.62, *p* < 0.01) for a skilled group of readers. Apostolidis et al. [54] also investigated anxiety but used a different questionnaire and found significant relations between the anxiety measure and bio signals (which included physiological arousal) for 80% of their participants. As the self-reported anxiety increased, participants’ SCR also increased. These significant results were found in studies that used the mean, amplitude, and latency of the EDA signal.

Another option is to address the *valence* of emotions and *arousal* through self-reports. As emotion is a multi-dimensional construct in most educational research, this approach has great potential [82]. Fox [49] used the Self-Assessment Manikin (SAM), which requires participants to mark their valence, arousal, and dominance levels on a 9-point Likert scale. They used a combined measure of self-reported valence, arousal, and dominance to relate to physiological arousal but found no significant correlations. A similar approach was used by Hussain et al. [62], but they used self-reported retrospective judgements as valence and arousal measures. They asked students to report low, medium, and high valence and arousal in a 3 × 3 grid in intervals of 10 s and tried to predict the self-reported valence and arousal with EDA. Their results indicate that students’ arousal and valence can be detected from EDA. Van Bruinessen et al. [69] did not find significant correlations between self-reported arousal and skin conductance level. They used a trait anxiety questionnaire of 10 items (4-point Likert scale) to address arousal. No significant results were found in studies that used mean arousal levels, which indicates a need for follow-up research with other features. The studies that found no significant results used retrospective self-report measures. Possibly, concurrent measurements at critical moments, such as feedback moments, can provide more insights into the valence-arousal approach to emotion.

Other studies investigate whether physiological arousal can *predict* self-reported emotions. Preliminary results show that SCL positively predicted the emotions anxiety and shame (*R^2^* = 0.25, *p* < 0.05; *R^2^* = 0.30, *p* < 0.05) [61].

**Behavioral Responses.** A few studies used behavioral responses of emotion in combination with EDA (*n* = 6); they used eye-tracking and facial expression detection. Mason et al. [65] used a Tobii T120 *eye-tracker* (sampling rate of 120 hertz) and found no significant interactions between eye-fixation (summed duration of all fixations during the first encounter with learning task) and EDA. A possible explanation is that they used the mean EDA of the whole learning session.

Another way to gain insight into the behavioral responses of participants is to detect their *facial expressions.* The analysis of facial expressions can focus on categorical emotions (as described by Ekman [19]) or on the dimensional features (e.g., as described by Pekrun [6]). FaceReader is a tool that is capable of both and is widely used in recent research. Harley et al. [46] used this tool to combine facial expressions with EDA and found an agreement rate of 60.1% between these data streams. The highest agreement was found between physiological arousal and sadness (64.3%) and neutral (70.8%). Not only discrete emotions can be detected from facial expressions, but also their valence, i.e., if a facial expression is positive or negative. Li and Lajoie [50] used FaceReader to address this. They used a valence intensity score (calculated by subtracting the sum of the intensity of negative emotions from positive emotions at each frame) and found that it is not a significant predictor of performance on an aviation task [50]. These non-significant results can be caused by the use of mean EDA during the whole task. Malmberg et al. [64] collected facial expressions during a collaborative learning task and used machine learning to estimate the valence score (support vector machine). In this study, they combined valence with periods of physiological synchrony, i.e., when students show similar EDA levels. Negative facial expressions occurred for 40% of the time during episodes of physiological synchrony, neutral expressions for 33%, and positive expressions for 22% of the time.

**Other Physiological Responses.** Fourteen studies used other physiological responses, such as skin temperature, EEG/EMG/ECG, heart rate, and mouse and chair pressure. Most of these studies did not report any results about the relation between EDA and the other measures but focused on relations with outcome measures, such as performance. Khan et al. [36] found significant links between physiological arousal and *skin temperature* across two semesters (*r* = 0.13, *p* < 0.05). *Heart rate* is combined with physiological arousal by Ahonen et al. [52], who reported a significant correlation in a collaborative setting.

More often, *multiple other physiological responses* are combined with EDA in clustering and affect detection. Sharma et al. [67] used multiple modalities to divide students into groups regarding their emotions (K-means clustering). They found that high EDA correlates with high emotional intensity measured by facial expression detection. This cluster of students also showed a high heart rate and low cognitive load. Another approach was used by Cooper et al. [57], who found that confidence, frustration, and excitement are best predicted by a combination of facial expression detection, mouse and chair pressure, and EDA (confidence: *R*^2^ = 0.06; frustration: *R*^2^ = 0.62; excitement: *R*^2^ = 0.56).

## 4. Discussion

This systematic review gave an overview of the literature regarding physiological arousal as measured by EDA in the learning and education context. The objectives of this review were: (1) to provide an overview of methodological aspects of EDA and investigate implicit guidelines and standards for EDA processing in educational research, (2) to examine existing empirical evidence of the interaction between physiological arousal as measured by EDA and both learning outcomes (I), and learning processes (II).

Results regarding the first objective showed that many different methodological approaches are used for measuring EDA in educational research. The first issue is the sampling rate of devices used. The most prevalent device to measure EDA is the Empatica, which has a sampling rate of 4 hertz. This sampling rate is considered sufficient for extended measurement periods, even though higher rates are recommended in the literature [3,72]. The Biopac and Biosemi devices have a sampling rate of 1000 hertz but are primarily used in laboratory settings and are less appropriate for use in classroom settings due to their size. Placement of electrodes on the fingers of the non-dominant hand is most promising for measuring EDA. Future research should use devices capable of measuring EDA in authentic settings with a sufficient sampling rate and high-quality signal to provide meaningful insights for educational practice. Thus, there is an opportunity for researchers to use wearable devices with a higher sampling rate in real-life settings, such as the Shimmer3 GSR+. However, more research is needed to establish the reliability and validity of measurements with these wearables.

The included studies employed different methods to process the EDA signal. In accordance with previous findings, the extraction of tonic and phasic components is most commonly used [4]. However, there is no consensus on which component to use in analyses: tonic, phasic, or both. Few studies discuss their rationale for using tonic or phasic components. Hence, we highly recommend that researchers justify their choices. Remarkably, nine studies did not report any information about the data cleaning and filtering of their EDA signal. It remained unclear whether authors did not process their EDA signal or simply did not report it, so it is recommended to report data cleaning procedures clearly for future studies. There was a considerable variation regarding baseline measures, with only half of the studies using a form of baseline measurement. Baseline activities, processing, and recording length varied among these studies. Most other application fields of EDA view baseline measurement as good practice because it signals non-responders early, i.e., participants with little to no variation in their EDA [35]. Therefore, we recommend that educational scholars to look for good practices in other fields and apply them in their research.

The same diffuse picture was found for the features extracted from EDA and the extraction methods. Most studies used the mean EDA over a specific period of time or during an activity. Due to large differences in learning activities, this period varied from 30 min to one-minute segments. Additionally, the mean EDA over an extended period of time can lead to overestimation or underestimation of EDA at critical moments. Using event-specific skin conductance responses could circumvent this problem, but researchers should pay close attention when determining which EDA response is linked to a specific event [30]. Many other features were extracted without indicating a rationale (see Table 3). It is essential for good practice that future research provide argumentation for the choice of features, so the learning and education field can develop guidelines and standards for EDA processing. Focusing on critical moments during learning can be useful, for example, when investigating the response to feedback. To analyze critical moments, a response window needs to be defined, which corresponds with the slow-moving nature of EDA [34]. In almost all of the included studies (24 out of 27), no information was given on whether a response window was used or its length. This is problematic because of the slow nature of the EDA signal, which shows variations only after a second [34]. Due to the large variety of features, there is a need for studies in the educational domain to assess the processing methods and evaluate their quality.

To conclude, the methodological aspects of measuring EDA in learning contexts differ largely. There are hardly any implicit standards found. Furthermore, we signal the usage of wearable devices with low sampling rates and no consensus on signal processing. Therefore, there is an urgent need for guidelines and standards for processing EDA data in educational research. An initial step would be to investigate the applicability of practices and tools of other research fields in educational research. These other fields use different tools to extract features from EDA: tools that use phasic drivers (e.g., SparsEDA [83]) and spectral indices (e.g., EDASymp [84] and TVSymp [85]). These tools have the potential to investigate EDA independent of time with time-varying analysis, which results in more sensitive EDA features [86]. After investigating these more robust practices and methods to extract EDA features, preliminary guidelines for the education and learning field can be drafted, and their quality should be investigated.

Methodological recommendations for educational researchers:

- Use devices capable of measuring EDA through electrodes placed on the fingers of the nondominant hand, in authentic settings, and with a sufficient sampling rate
- Justify choices for using tonic or phasic components
- Report data cleaning and filtering procedures clearly
- Look for good practices regarding baselines in other scholarly fields
- Provide argumentation for choice of features
- Define an appropriate response window
- Need for guidelines and standards for EDA processing

With respect to empirical findings in the second objective, half of the studies examined the relationship between physiological arousal as measured by EDA and learning outcomes. The majority found significant relations between students’ test and task performance and EDA. Moreover, learning gains and changes in EDA were also related. Results are hard to compare due to different ways of data processing, as tonic and phasic components were used as well as different features, such as frequency of SCR peaks and mean EDA. Additionally, the usage of different tasks and tests complicates evaluation. Studies also differed in their theoretical frameworks, using (collaborative) engagement, stress, affective states, and anxiety theories. Hence, no overarching conclusions of the relation between physiological arousal as measured by EDA and learning outcomes can be drawn due to the diverse nature of the studies.

Regarding measurements of the learning process, we found that 12 studies focused on unimodal approaches to investigate changes in EDA during learning. Indeed, most studies found changes during the learning process, but the direction of these changes remained inconclusive as both decreases and increases of EDA during learning were found. This indicates that EDA varies during the learning process, but we do not know which contextual and/or personal characteristics explain these changes. Two studies further examined changes after specific events, e.g., after running a code in a programming task and after self-reported emotion, and found correlations with learning outcomes [46,52]. Hence, there is potential in investigating EDA changes at these critical moments to gain a deeper understanding of students’ emotions during learning. 

We found that most included studies used multimodal data streams. In seven of them, no mutual relations between these data streams and EDA were examined at all, but they related EDA to another outcome measure instead, such as performance. Most studies that did relate EDA to other multimodal data streams examined experiential responses with self-reports, but there was no clear agreement in their results. As discussed in the introduction, dimensional models prescribe the measurement of emotions in terms of valence and arousal. We indeed found two studies using self-reports for that purpose. Valence and arousal can be detected from physiological signals, and they can predict self-reported emotions. Moreover, most studies focused on outcome measures and not on critical moments. It would be highly valuable to investigate what happens at a detailed level, such as when a student receives feedback, so future research can provide meaningful insights into critical moments during learning. Valence has the potential to be a valuable addition to these critical moments by providing a direction to the EDA data.

Six studies examined behavioral responses through facial expression detection or eye-tracking. Only one study examined the relation between EDA and eye-tracking and found no significant relation. A possible explanation is that the eye-tracking features used in this study are not fine-grained enough, as fixation rates are summed up during the first encounter with the learning task. With regard to facial expressions, both specific emotions and their valence (positive or negative) were inferred from the data. Studies that used valence produced inconclusive results, from no significant relations with EDA to the occurrence of negative facial expressions during episodes of similar EDA levels in a group for 40% of the time. The non-significant results can be caused by the use of mean EDA during a whole task when a more fine-grained EDA feature could possibly lead to significant findings. As facial expression detection as a measure of behavioral responses seems promising in its relation to fine-grained EDA features, it is interesting to investigate this further.

Half of the studies also used other physiological arousal measurements: heart rate, EMG, ECG, EEG, and skin temperature. It is notable that most of these studies did not analyze the relation of these physiological measurements with EDA. The two studies that did found a significant correlation between EDA and skin temperature and EDA and heart rate, showing the potential of combining these data streams. Multiple other physiological measurements are also combined with EDA to cluster students, and studies reported that high EDA levels correlate with high emotionally intense facial expressions and high heart rate. Self-reported emotions were also predicted by combining EDA with facial expression detection and mouse and chair pressure. More research is needed into the connections between EDA and other physiological measures at critical moments, to prevent missed opportunities of not connecting the EMG, ECG, EEG, heart rate, and skin temperature data to EDA as happened in previous studies.

Empirical recommendations for educational researchers:

- Potential in investigating EDA changes at critical moments during the learning process
- More research needed into experiential measures regarding valence
- Facial expression detection seems promising to connect EDA with behavioural measures
- Analyze the relation between EDA and other physiological measures (EEG, ECG, EMG, heart rate, and skin temperature)
- Potential in investigating combinations of EDA and experiential, behavioural, and other physiological measures at critical moments

This summary of results shows a gap in the literature regarding multimodal data streams to combine experiential, behavioral, and physiological responses. Previous studies using these multimodal data streams do not always analyze the mutual relations between them but only look at outcome measures such as performance. The combination of experiential, behavioral, and physiological responses has a great potential to understand critical moments. Connecting different data streams to capture these responses allows us to apply measurements in a fine-grained manner and examine interrelations in detail.

## 5. Conclusions

Developments in measuring EDA are on the rise, making it easier to gain insight into physiological arousal and consequently emotion in authentic learning settings. This review showed a wide variation in processing steps taken by researchers, which points to a need to develop guidelines and standards in the field concerning practices and reporting. An opportunity for future research is to design studies that investigate and explain fluctuations in EDA. Approaches focusing on critical moments during the learning process and relating these to EDA have great potential [3,27,32]. Moreover, using multimodal data streams to measure experiential, behavioral, and physiological responses helps gain an even deeper insight into learning. We see ample opportunities for educational researchers to collaborate with other scholarly disciplines in developing guidelines and exploring learning processes at a deeper level.

## Figures and Tables

**Table 1:** Study Characteristics.

Ref.	Participants ^1^	Age ^2^	*n*	Type of Task	Domain	Study Type ^3^
[52]	University students	23.00	38	Collaborative Programming	Computer sciences	Case study
[53]	University students + adults	-	11	VR: Virtual patient scenario	Medicine	Experiment
[54]	University students	26.04 (2.30)	15	Educational game virtual patient	Medicine	Experiment
[55]	High school students	-	21	Building a bridge and a tower	Physics	Experiment
[39]	Primary school students	11.60 (0.54)	214	Inquiry-based learning lessons	Sciences	Case study
[56]	University students + adults	18–45	24	VR: problem-solving task	Problem- solving	Case study
[57]	High school students	-	35	ITS: geometry tasks	Geometry	Experiment
[58]	Adults	25.87 (3.85)	15	Educational game: stakeholder management	Project management	Experiment
[49]	University students	23.50 (6.57)	70	Vocabulary training	Language	Experiment
[59]	Primary school students	7.50 (0.47)	104	Geometry tasks & physical learning	Geometry	Experiment
[60]	Students	-	38	Programming tasks	Computer sciences	Case study
[46]	University students	21.00 (1.90)	67	ITS: human circulatory system tasks	Biology	Case study
[61]	University students	24.30 (3.50)	37	Diagnostic reasoning tasks	Medicine	Experiment
[40]	University students	20.63 (2.13)	61	Electrical circuits troubleshooting	Physics	Experiment
[62]	University students	18–30	20	ITS: physics, computer literacy, critical thinking tasks	Physics	Experiment
[63]	University students	19–20	18	E-learning: mathematics & electric circuit tasks	Mathematics & physics	Experiment
[36]	University students	-	76	Exam	Engineering	Experiment
[50]	University students	24.37 (5.81)	19	Aviation training	Aviation	Experiment
[64]	High school students	17.4 (0.67)	48	CSCL: design a healthy breakfast	Biology	Case study
[65]	Primary school students	12.37 (0.55)	48	Reading comprehension task	Language	Experiment
[66]	Adults	21–34	39	Reading task	Language	Experiment
[33]	High school students	16–17	24	Online exam	Physics	Case study
[67]	University students	19.24 (0.83)	32	Programming questions	Computer sciences	Case study
[68]	University students	23.20 (4.07)	95	Test	Mathematics	Experiment
[69]	Adults	33.10 (13.40)	75	Educational video’s	Medicine	Experiment
[70]	University students	18–20	18	Workshop design	Design	Experiment
[71]	University students	-	7	Engineering problems	Engineering	Experiment

^1^ Adults refer to all adults with no enrollment in a specific form of education (such as university). ^2^ M (SD) or range, - means no information is given, VR = Virtual Reality, ITS = Intelligent Tutoring System, CSCL = Computer Supported Collaborative Learning. ^3^ We defined a case study as an in-depth exploration and an experiment as a study in which specific relations and hypotheses are tested in an experimental setting.

**Table 2:** Methodological Aspects per Included Study.

Ref.	Device	Processing		Baseline		
		Filtering	Cleaning	Activity	Length	Usage
[52]	Shimmer3 GSR+	-	Interpolation Normalization	Video	7 min	-
[53]	Empatica E4	-	-	Learning session	-	Average in plots
[54]	Self-assembled	Low-pass filter	-	-	-	-
[55]	ProComp Infiniti	-	Manual and visual	Different tasks & video	22 min	Calculate difference score
[39]	Empatica E3	High and low-pass filter	-	-	-	-
[56]	Empatica E4	-	Machine learning	-	-	-
[57]	MIT sensor	-	-	-	-	-
[58]	Electrodes Ag/AgCl filled	Low-pass filter & down-sampling	-	No specific activities	5 min	Mean baseline as covariate
[49]	BioSemi Active 2	Down-sampling	-	Resting time & practice video’s	5 min	Segmenting signal
[59]	BodyMedia Core	Non-specified	Accelerometer	-	-	-
[60]	Not specified	-	-	-	-	-
[46]	Q-Sensor 2.0	-	-	No specific activities	10–15 min	Correction for normalization
[61]	Q-Sensor 2.0; Biopac	-	-	No specific activities	2–5 min	Correction for normalization
[40]	Empatica E4	-	-	Learning session	-	Used in analysis
[62]	Biopac	-	-	-	-	-
[63]	Biopac	High-pass	Normalization	-	-	-
[36]	Empatica E4	-	Accelerometer L2 norm calculation	-	-	-
[50]	BioNomadix	Non-specified	Non-specified	-	-	-
[64]	Empatica E3	Adaptive Gaussian filter	Manual, visual Normalization	-	-	-
[65]	ProComp Infiniti	-	Normalization	Watching video & learning session	4 min	Calculate difference score
[66]	Biosemi Active 2	Down-sampling	-	No specific activities	-	Analysis
[33]	Empatica E4	No processing	No processing	-	-	-
[67]	Empatica E4	-	-	-	-	-
[68]	Empatica E4	-	-	Breathing exercise	5 min	Analysis
[69]	Biopac	Low-pass filter	-	Watching video	30 s	Comparing to baseline
[70]	Empatica E3	-	Normalization	No specific activities	-	-
[71]	Empatica E3	-	Accelerometer Normalization	-	-	-

- means no information is given.

**Table 3:** Features and Feature Extraction Methods.

Ref.	Features	Extraction Features in Segments/Whole Session (Time) ^1^	Feature Extraction Methods
[52]	Standardized SCR & SCL score	Time segment around event (20 s)	Ledalab
[53]	Mean	Task segment (varying)	-
[54]	Mean	Task segment (varying)	Manual
[55]	Standardized SCL score	Time segment (2 min)	Biograph Infiniti
[39]	Mean	Whole learning session (45–60 min)	Manual
[56]	Mean, SD, min, max, percentiles	Time segment (1 min)	cvxEDA-tool
[57]	Mean, SD, min, max	Time segment around event (90 s)	-
[58]	Mean	Time segment (1 min)	Ledalab
[49]	Mean	Task segment (40 s)	Ledalab
[59]	Mean	Whole learning session (2 h)	Manual
[60]	Standardized SCL score	Time segment around event (5 s)	Ledalab
[46]	Mean, range	Time segment around event (10 s)	Augsburg toolbox
[61]	Number of SCR peaks, Standardized SCL score	Whole learning session (2.5 h)	-
[40]	Mean	Task segment (varying)	-
[62]	-	Time segment (10 s)	Augsburg toolbox
[63]	Mean	Time segment (1 min)	-
[36]	Mean	Whole learning session (-)	Ledalab
[50]	Mean	Task segment (-)	Neurokit
[64]	Number of SCR peaks, Frequency of SCR peaks	Time segment (1 min)	Ledalab
[65]	Mean	Task segment (4 min)	-
[66]	Amplitude sum of SCR peaks, Latency of SCR peaks	Whole learning session (1 h)	Ledalab
[33]	Number of SCR peaks, Onset of SCR peaks	Time segment (1 min)	Ledalab
[67]	Mean	Task segment (varying)	-
[68]	Frequency of SCR peaks	Time segment (1 min)	Ledalab
[69]	Mean, Number of SCR peaks	Task segment (59–79 s)	Acqknowledge
[70]	Mean	Whole learning session (75 min)	Manual
[71]	Mean	Whole learning session (-)	-

- means no information is given. ^1^ Extraction of features from the EDA signal was done in segments or over the whole learning session. Task segments are based on the time spent on a task. Time segments are specific periods of time, which also can be initiated around a specific event (such as entering an answer). Whole learning session: EDA features are extracted from the whole track, which consists of multiple tasks.

**Table 4:** Empirical Aspects Per Included Study.

Ref.	Interaction EDA— Learning Outcomes	Unimodal	Multimodal			
		Interaction EDA— Learning Process	Experiential	Behavioral	Other	Multimodal Results
[52]	Differences before and after pass and fail events	Multimodal	-	-	Heart rate	Correlation between heart rate and SCR
[53]	-	Increasing EDA during learning	-	-	Heart rate, EEG	No results
[54]	-	Variations in EDA during segments of learning	Self-report anxiety	-	EEG	Correlation between EDA and self-report (no results EDA—EEG)
[55]	-	U-shaped EDA during learning	x	x	x	x
[39]	Positive correlation between science knowledge and changes in EDA	Increasing EDA during learning	x	x	x	x
[56]	Classifier with EDA to indicate Aha! Moment (83.66%)	-	-	-	Heart rate	No results
[57]	-	Multimodal	Self-report emotion	Facial expression detection	Mouse & chair pressure	Predicting emotions during learning
[58]	Tonic EDA predicts learning gain	-	-	-	EMG & ECG	No results
[49]	Change in tonic EDA over time predicts performance		Self-report emotion	-	Heart rate, HRV, ECG	No significant relations
[59]	-	Higher EDA in physical learning	Self-report valence	-	Skin temperature	No results
[60]	Bigger learning gains when SCR after specific event	-	Self-report engagement	-	-	No results
[46]	-	Multimodal	Self-report emotion	Facial expression detection	-	Relations between modalities
[61]	Phasic EDA can predict learning	Multimodal	Self-report emotion	-	-	SCL positively predicts anxiety and shame
[40]	No association EDA and performance	No difference baseline EDA and EDA during task	Self-report worry	-	-	No results
[62]	-	Multimodal	Self-report emotion	-	EMG & ECG	Predicting self-report with EDA
[63]	-	Decreasing EDA during learning (SCL)	-	-	ECG	No results
[36]	Positive correlation EDA and performance	Multimodal	-	-	Skin temperature	Positive correlation skin temperature and EDA
[50]	Phasic EDA can predict performance	-	Self-report	Facial expression detection	-	-
[64]	-	Multimodal	-	Facial expression detection	-	Negative (40%), neutral (33%), positive facial expressions (22%)—physiological synchrony
[65]	High arousal relates to low performance	-	Self-report emotional problems	Eye-tracking	-	No significant relations
[66]	-	EDA oral reading > silent reading (skilled readers)	Self-report anxiety	-	Heart rate	Positive correlation self-report anxiety and EDA (no results heart rate)
[33]	Frequency of arousal periods correlates with performance	Mean 60% low arousal, 24% medium, 17% high	x	x	x	
[67]	-	Multimodal	-	Facial expression detection, eye-tracking	Heart rate, EEG, skin temperature	High EDA correlates with high emotion, high heart rate, low mental workload, and memory load
[68]	No association EDA and performance	Multimodal	Self-report Anxiety	-	-	No significant relations
[69]	-	Increasing SCL during learning compared to baseline (not for SCR)	Self-report arousal	-	Heart rate	No significant relations
[70]	-	Increase in EDA during learning (more when active learning)	Self-report emotion	-	-	Correlation between EDA and negative emotions and positive emotions
[71]	No significant relation EDA and performance on tasks	Decrease EDA in two of three tasks	Self-report emotion	-	-	Correlation between EDA and self-reported emotion before the task

x means no multimodal approach, - means no information is given.
